# Comparison of *in vitro* Neuronal Differentiation Capacity Between Mouse Epiblast Stem Cells Derived From Nuclear Transfer and Naturally Fertilized Embryos

**DOI:** 10.3389/fnmol.2018.00392

**Published:** 2018-10-30

**Authors:** Tong Li, Yi Zheng, Yan Li, Danna Ye

**Affiliations:** ^1^State Key Laboratory of Ophthalmology and Vision Science, School of Ophthalmology and Optometry, Eye Hospital, Wenzhou Medical University, Wenzhou, China; ^2^Department of Reproductive Medical Center, The First Affiliated Hospital of Wenzhou Medical University, Wenzhou, China

**Keywords:** mEpiSCs, nuclear transfer, pre-gastrulation stages, *in vitro*, neuronal differentiation, neurogenesis delay

## Abstract

Somatic cell nuclear transfer (SCNT) can give rise to fertile adults, but the successful perinatal and postnatal developmental rates are inefficient, including delayed developmental behaviors, and respiratory failure. However, the molecular and cellular mechanisms remain elusive. Mouse epiblast stem cells (mEpiSCs) from E5.5-6.5 epiblasts share defining features with human embryonic stem cells (hESCs), providing a new opportunity to study early mammalian development *in vitro.* In this study, mEpiSCs were established from naturally fertilized mouse embryos (F-mEpiSCs) and SCNT mouse embryos (NT-mEpiSCs). Also, the *in vitro* neuronal differentiation capacity of F-mEpiSCs and NT-mEpiSCs was compared. Morphology analysis showed less and smaller neurospheres formation and lower percentage of early neurons generation in NT-mEpiSCs. The immunocytochemical analysis and altered mRNA expression levels of the neuronal markers in differentiated cells further confirmed that neurogenesis was slower in NT-mEpiSCs than in F-mEpiSCs. Moreover, neuronal differentiation capacity was correlated with the basal expression levels of Atox1 and Vinculin but not Brachyury and Otx2, emphasizing that developmental aberrations in neurogenesis were associated with the NT technique but not random variations between clones. This study provided an important *in vitro* platform using mEpiSCs to study early epigenetic and developmental processes associated with neurogenesis.

## Introduction

Somatic cell nuclear transfer (SCNT) is an animal cloning technology that can give rise to fertile offspring. It provides an important research tool for studying reprogramming, genomic imprinting, genomic reprogramming, and embryonic development (Niemann and Lucas-Hahn, 2012). The successful cloning of Macaque monkey recently (Liu et al., 2018) also provides potential for human disease modeling and drug development. However, typical low success rates and high incidence of developmental abnormalities are major obstacles to its widespread use (Niemann and Lucas-Hahn, 2012; Qiu et al., 2017). Over the years, cloning efficiency has significantly improved, but only 3–5% of the transferred embryos develop to term (Petersen et al., 2008) and often die due to pre- and postnatal developmental aberrations (Young et al., 1998). Importantly, it is widely accepted that such abnormalities are caused by insufficient or faulty reprogramming of the transferred somatic cell nuclei. A promising mechanism responsible for the developmental aberrations in nuclear transfer and reprogramming is the disruption of genomic imprinting (Dean et al., 2001). Indeed, methylation reprogramming of the genome is a key feature during early mammalian development because it orchestrates the changes in gene expression required for the timing of first cell division, compaction, blastocyst formation, expansion, and hatching (Feng et al., 2010). Most imprinted genes have key roles in fetal and placental growth and differentiation, and the expression of several such genes, such as *ndn* and *xist*, was found to be altered in cloned animals (Mann et al., 2003). The fine relationship between reprogramming and genomic imprinting is further supported by the altered expression of imprinted genes, such as *peg3*, *maoa*, and *peg*, in aborted cloned calves (Liu et al., 2008).

The successful derivation of mEpiSCs from post-implantation embryos and the realization that these cells share defining features with human hESCs provide a new opportunity to study early mammalian development *in vitro* (Brons et al., 2007; Tesar et al., 2007; De Los Angeles et al., 2012), especially for human development and disease modeling. In contrast to naïve mouse embryonic stem cells (mESCs), mEpiSCs, and hESCs are considered as primed pluripotent stem cells because the later cells undergo random X-inactivation and require similar signaling pathways governing self-renewal (De Los Angeles et al., 2012). mEpiSCs and hESCs share a similar epigenetic state and have monoallelic expression of most imprinted genes, which tend to lose imprinting in mESCs (Sun et al., 2012). However, transcriptional regulation and marker expression of mEpiSCs are different in some respects from hESCs. For instance, hESCs express the stem cell marker REX1, similarly to mESCs, and mEpiSCs express the *Fgf5*, which is absent in hESCs and mESCs (Tesar et al., 2007). The inaccessibility to hESCs and the similar epigenetic states of mEpiSCs and hESCs reinforce the importance of mEpiSCs instead of hESCs in the study of early mammalian development *in vitro*.

Indeed, genetic and epigenetic alterations that occur during embryonic development are evident in the late epiblast stage, consistent with the divergent gene expression profiles of mEpiSCs derived from NT (NT-mEpiSCs) and naturally fertilized embryos (F-mEpiSCs), but not mESCs (Maruotti et al., 2010; Jouneau et al., 2012). The transcripts mostly altered in NT-mEpiSCs are all imprinted genes, including *Snrpn/Snurf, Ndn, Impact*, and *Peg3* (Maruotti et al., 2010), and their dysregulation may at least partly explain the mortality observed just after implantation in NT embryos (Jouneau et al., 2006). Furthermore, most of the imprinted genes expressed in the brain were strongly downregulated in NT-mEpiSCs, thereby having a crucial role in neuronal development. This suggests that such genetic or epigenetic changes can lead to abnormal neuronal development. Based on this hypothesis, this study aimed to establish the F-mEpiSCs and NT-mEpiSCs, compare their *in vitro* neuronal differentiation capacity, and figure out the underlying reasons for the difference.

## Materials and Methods

All reagents were purchased from Sigma–Aldrich unless otherwise indicated.

### Derivation and Maintenance of Mouse Epiblast Stem Cells

All animal care and surgical interventions were undertaken in strict accordance with the approval of the Wenzhou Medical University Animals Ethics committee. Further, 8- to 10-week-old B6D2F1 (C57BL/6N × DBA/2) mice were used to produce naturally fertilized and NT embryos as previously described (Wakayama et al., 1998). mEpiSC lines were established and maintained as previously described (Brons et al., 2007; Tesar et al., 2007). Briefly, pre-gastrulation stage (E5.5) mouse embryos from natural fertilization and NT were treated with cell dissociation buffer for 20 min at 4°C. They were dissected and separated into small pieces in HEPES-buffered medium using glass needles. The isolated epiblast fragments were then placed in completely defined medium (CDM) supplemented with 20 ng/mL Activin A (R&D Systems Inc.) and 12 ng/mL basic fibroblast growth factor (bFGF; R&D Systems Inc.) at 37°C with 5% humidified CO_2_. CDM consisted of 50% IMDM (Gibco), 50% F-12 (Gibco), 5 mg/mL BSA (Europa Bioproducts), 1% lipid 100 × (Gibco), 450 μM monothioglycerol, 7 μg/mL insulin, and 15 μg/mL transferrin. The cells were incubated with 5 mg/mL collagenase II every 4–5 days and manually passaged to new serum-coated plates.

### Spontaneously Differentiation

Embryoid body (EB) formation was performed by transferring mEpiSCs to low attachment plates (Corning) in DMEM/F12 containing 20% knockout serum replacement (Gbico), 1% NEAA (Gibco), 2 mM L-glutamine (Gibco), 0.1 mM 2-mercaptoethanol (Gibco), and 1% PenStrep (Gibico). After 7 days, the EBs were plated on 0.5 μg/cm2 vitronectin (Gibco) coated dishes and cultured in DMEM supplemented with 10% FBS, 2 mM L-glutamine and 1% Pen/Strep for up to 3 weeks.

### Neuronal Differentiation

mEpiSCs were differentiated into early neurons by employing a protocol previously used to differentiate hESCs and mESCs (Suter et al., 2009; Martinez et al., 2012) with minor modifications. For neuronal differentiation, mEpiSCs were scraped and cultured in suspension on bacterial Petri dishes in neural differentiation medium 1 (composed of F-12, 2% N2, 1% B27, 1% NEAA, and 10 ng/mL bFGF) to form neurospheres (Cho et al., 2008). After 4 days, spheres were dissociated into single cells using 0.05% (w/v) trypsin/0.02% (w/v) EDTA in phosphate-buffered saline (PBS) and plated onto poly L-ornithine/laminin-coated plates in neural differentiation medium 2 (composed of F-12, 2% N2, 1% B27, and 1% NEAA) for neuron generation. Where indicated, the cells were counted, and a fixed number of cells were plated (1.5 × 10^4^ cells/cm^2^).

### cDNA Synthesis and Quantitative Real-Time Polymerase Chain Reaction

Total RNAs were prepared according to the manufacturer’s protocol using the RNeasy Mini Kit (Qiagen), followed by reverse transcription using Superscript II Reverse Transcriptase (Invitrogen). Real-time polymerase chain reaction (PCR) was performed in SYBR Green JumpStart Taq ReadyMix in a total volume of 10 μL using the following cycling parameters: 94°C for 3 min initial denaturation followed by 40 cycles of 95°C for 5 s, 60°C for 15 s, and 72°C for 30 s. The melting curve analysis confirmed the specificity of primers and ruled out primer–dimer artifacts. GAPDH was used as endogenous control. The primer sequences used in this study are listed in Table 1.

**Table 1:** List of primers for quantitative RT-PCR.

Symbol	Primers	Reference
GAPDH	F: GGAGCGAGACCCCACTAACATC	NM_001289726.1
	R: CTCGTGGTTCACACCCATCAC	NM_008084.3
Rex1/zfp42	F: CCAGGTTCTGGAAGCGAGTT	NM_009556.3
	R: AGTAATGAGCTCGCCCCAAC	
Nanog	F: CGGTGGCAGAAAAACCAGTG	NM_028016.3
	R: AAGGCTTCCAGATGCGTTCA	NM_001289828.1
		NM_001289830.1
		NM_001289831.1
Oct3/4	F: CAGACCACCATCTGTCGCTT	NM_013633.3
	R: CTCCACCTCACACGGTTCTC	NM_001252452.1
Fgf5	F: AGTCCCAGCCCAGTGAAGT	NM_010203.5
	R: TTCCACACGTGTAGGCACAG	NM_001277268.1
Pax6	F: CTTCAGTACCAGGGCAACCC	NM_001244198.2
	R: CGCATCTGAGCTTCATCCGA	NM_001244200.2
		NM_013627.6
		NM_001244201.2
		NM_001244202.2
		NM_001310144.1
		NM_001310145.1
		NM_001310146.1
Tuj1	F: TGAGGCCTCCTCTCACAAGT	NM_023279.2
	R: GTCGGGCCTGAATAGGTGTC	
Sox1	F: CAGCCCCATCTCCAACTCTC	NM_009233.3
	R: CGACTTGACCAGAGATCCGA	
Map2	F: AGACCTTCCTCCATCCTCCC	NM_001310634.1
	R: GCCACTTTTTCCTGCTCTGC	NM_008632.2
		NM_001039934.1
Atox1	F: GGGAGGAGTGGAGTTCAACA	NM_009720.3
	R: CTGTTTTGTTGAGGGTTGCCA	
Vinculin	F: TGGTCTAGCAAGGGCAATGA	NM_009502.4
	R: CTCGTCACCTCATCAGAGGC	
Brachyury	F: GTTCCCGGTGCTGAAGGTAA	NM_009309.2
	R: GCGAGTCTGGGTGGATGTAG	
Otx2	F: GGGCTGAGTCTGACCACTTC	NM_001286481.1
	R: ACAGAGCTTCCAGAACGTCG	NM_001286482.1
		NM_144841.5
		NM_001286483.1
		NM_001360225.1
		NM_001360226.1

### Immunofluorescence

Undifferentiated mEpiSCs and differentiated cells were fixed in 4% paraformaldehyde for 15–20 min, permeabilized with 0.2% (v/v) Triton X-100 in PBS, and blocked with 3% BSA and 0.5% Tween 20 in PBS. The cells were subsequently incubated with primary antibodies shown in Table 2 at 4°C overnight. Then, they were treated with the secondary antibodies Alexa 488 donkey anti-rabbit immunoglobulin G (IgG) (Molecular Probes, A21206, 1:1000) or Alexa 568 goat anti-mouse IgG (Molecular Probes, A11031, 1:1000) for 1 h at room temperature in the dark. To visualize the nuclei, the cells were counterstained with 4′, 6′-diamidino-2-phenylindole (DAPI) for 5 min and examined using a fluorescence microscope (Leica DMi8 with Leica DFC450 camera). Positive cells were quantified by counting cells showing positive staining divided by the total number of cells counterstained using DAPI.

**Table 2:** List of primary antibodies for immunofluorescence.

Symbol	Species	Company	Dilution
Nestin	Mouse	Invitrogen, MA1-110	1:400
Pax6	Rabbit	Invitrogen, 42-6600	1:400
NeuroD1	Rabbit	Abcam, ab60704	1:400
Tuj1	Mouse	Abcam, ab78078	1:1000
α-SMA	Rabbit	Abcam, ab5694	1:400
AFP	Mouse	Abcam, ab3980	1:400
Nanog	Rabbit	Peportech, 500-P236-100	1:400
SSEA1	Mouse	Invitrogen, MA1-022	1:400
Oct3/4	Mouse	Santa Cruz, sc-5279	1:200
GFAP	Mouse	Chemicon, MAB3402X	1:400

### Flow Cytometry

Neuronal differentiated cells were harvested and dissociated into single cells by trypsinization using 0.05% (w/v) trypsin/0.02% (w/v) EDTA in PBS. Then, 0.2 × 10^6^ cells were fixed in 4% paraformaldehyde for 15–20 min at room temperature and permeabilized with 0.1% (v/v) Tween 20 in PBS for 20 min. The cells were incubated in blocking buffer (10% fetal bovine serum in PBS) for 15 min, pelleted by centrifugation at 350 g for 5 min at 4°C, and incubated in 1% BSA in PBS containing anti-neuroD1 antibody (Abcam, ab60704) for 1 h at 4°C. They were washed and incubated with 1% BSA in PBS containing Alexa 488 donkey anti-mouse I gG antibody (Molecular Probes, A21202, 1:1000) for 1 h at 4°C, washed, and resuspended in PBS for flow cytometry analysis. Negative control cells were treated similarly except for the primary antibody staining. Mouse neuroblastoma Neuro-2A cells (ATCC CCL131) served as positive control. Data were acquired for a minimum of 10 × 10^3^ events per sample using the FC500 flow cytometer (Beckman Coulter Inc.) and analyzed using the FlowJo software (TreeStar Inc.).

### Statistical Analysis

All data represented three independent experiments, unless indicated otherwise. They were analyzed using one-way analysis of variance followed by Tukey’s *post hoc* tests using GraphPad Prism 6. A *P-*value < 0.05 were considered statistically significant.

## Results

### Derivation and Characterization of mEpiSCs From Naturally Fertilized and NT Embryos

**FIGURE 1:**
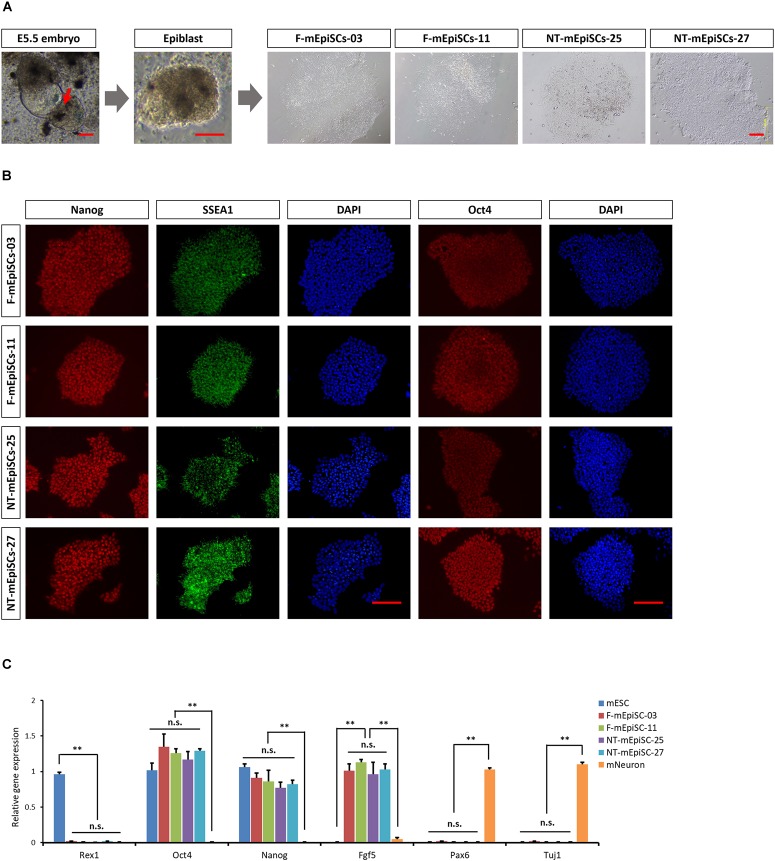
Derivation and characterization of mouse EpiSCs from *in vivo* fertilization and NT embryos. **(A)** Mouse embryo at E5.5 stage, isolated epiblast layer, and morphology of established F-mEpiSCs and NT-mEpiSCs. **(B)** Immunofluorescence staining for Nanog, SSEA1, and Oct4 in F-EpiSCs and NT-EpiSCs; nuclei are shown using DAPI. **(C)** Gene expression analysis by qPCR for EpiSC-specific markers, pluripotency markers, and neural marker in four selected mEpiSCs. Scale bar: 100 μm; n.s., *P* > 0.05; ^∗∗^*P* < 0.01.

Epiblasts from pre-gastrulation stages (E5.5) mouse embryos were dissected after treatment with cell dissociation buffer. They formed colonies of flat and compact cells with high nucleocytoplasmic ratios attached to the serum-coated plates. The morphology showed no difference between cell lines derived from different embryos. Two mEpiSC lines established from naturally fertilized embryos (F-mEpiSCs-03 and F-mEpiSCs-11) and two mEpiSC lines established from SCNT embryos (NT-mEpiSCs-25 and NT-mEpiSCs-27) with same genetic background were selected for this study (Figure 1A). F-mEpiSCs and NT-mEpiSCs were characterized using immunocytochemistry and quantitative real-time PCR (qPCR) methods. Immunostaining showed that both F-mEpiSCs and NT-mEpiSCs expressed the pluripotency markers Oct 4, SSEA1, and Nanog (Figure 1B). qPCR demonstrated that the expression levels of Oct4, Nanog, and Fgf5 were similar in all selected mEpiSCs. F-mEpiSCs, and NT-mEpiSCs did not express ESC-specific marker Rax1 and neural-specific markers Pax6 and Tuj1 (Figure 1C). Since mEpiSCs are known to express some early differentiation markers (Tesar et al., 2007; Frohlich et al., 2013), it was confirmed that Pax6 and Tuj1 were absent in four mEpiSC lines. Furthermore, spontaneously differentiation followed by immunostaining analysis of the endodermal marker α-fetoprotein (AFP), the mesodermal marker alpha smooth muscle actin (α-SMA) and the ectodermal marker β-III tubulin (Tuj1) confirmed the ability of F- and NT- mEpiSCs to form embryoid body and differentiate into all three germ layers (Supplementary Figure S1). These results indicated the successful establishment of F-mEpiSC and NT-mEpiSC cell lines for subsequent experiments.

### Neuronal Differentiation of mEpiSCs *in vitro*

**FIGURE 2:**
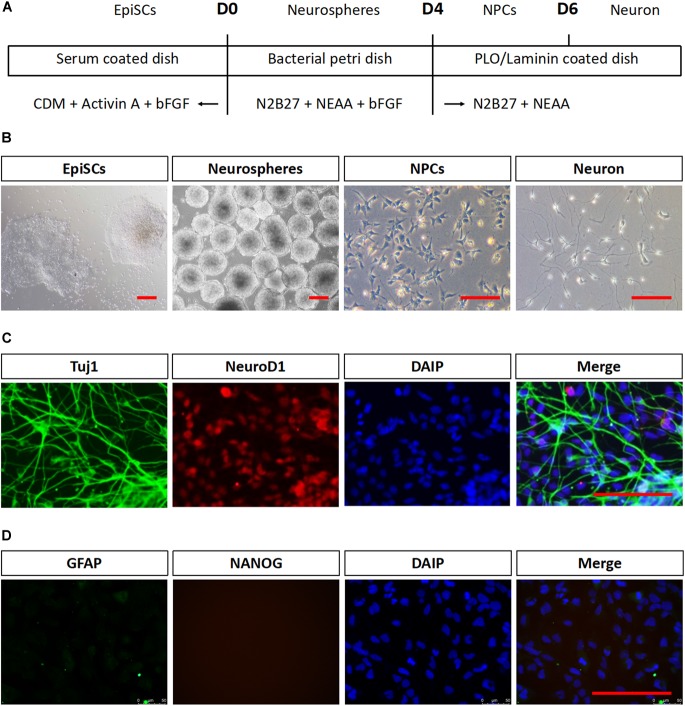
Neuronal differentiation *in vitro* of mEpiSCs. mEpiSCs were allowed to form neurospheres in suspension, which were dissociated and plated on poly L-ornithine/laminin-coated plates, resulting in early neural cells. **(A)** Scheme depicting the differentiation experiment workflow. **(B)** Morphology of mEpiSCs colonies, neurospheres formed following incubation in differentiation media for 4 days, neural progenitor cells, and early neurons. **(C,D)** Immunofluorescence staining for Tuj1, NeuroD1, GFAP, and Nanog in mEpiSCs-derived early neurons. Tuj1 and NeuroD1 were positive, whereas GFAP and Nanog were negative. Scale bar: 100 μm.

mEpiSCs were dissociated and allowed to form neurospheres in suspension for 4 days in neuronal differentiation media to test whether mEpiSCs had neural differentiation capacity. Then, they were dissociated into single cells and plated on poly L-ornithine/laminin-coated plates for further maturation (Figures 2A,B). Neurospheres were formed after 2–3 days, and by day 4, they attained a diameter of about 100 μm and distinct neurite-like internal structures were evident (Figure 2B). Two days following cell plating in poly L-ornithine/laminin-coated plates, the cells became smaller and bright, and axons could be observed (Figure 2B). Immunostaining was performed for the neuronal markers NeuroD1 and Tuj1, astrocyte marker GFAP, and pluripotency marker Nanog. The cells were positive for both markers as shown by NeuroD1 nuclear staining and typical Tuj1-positive network of axons, negative for GFAP and Nanog, supporting the successful generation of early neurons (Figures 2C,D).

### Quantification of Neurosphere Formation From F-mEpiSCs and NT-mEpiSCs

**FIGURE 3:**
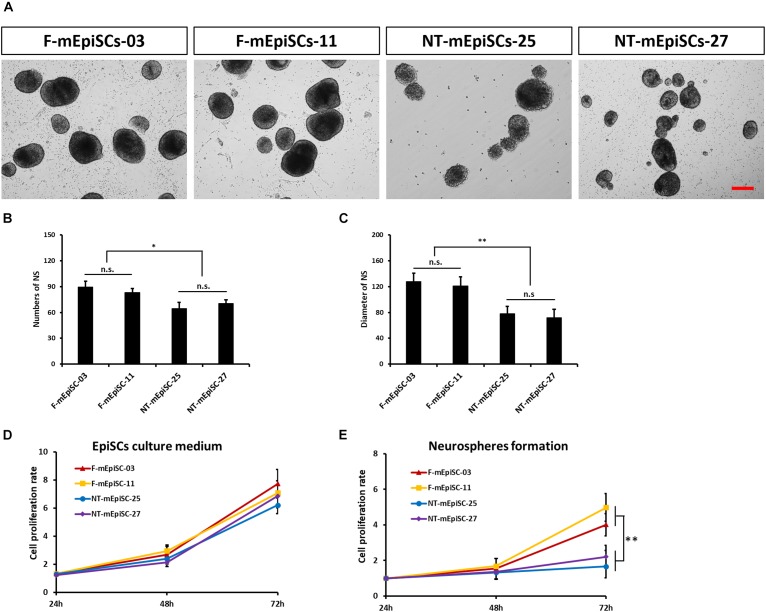
Quantification of neurosphere formation from F-mEpiSCs and NT-mEpiSCs. **(A)** Morphology of neurospheres formed from F-mEpiSCs and NT-mEpiSCs. **(B,C)** Numbers and average diameter of neurospheres derived from F-mEpiSCs and NT-mEpiSCs. **(D)** Cell proliferation rate of F-mEpiSCs and NT-mEpiSCs cultured in EpiSC culture medium. **(E)** Cell proliferation rate of F-mEpiSCs and NT-mEpiSCs during neurosphere formation. Scale bar: 100 μm; n.s., *P* > 0.05; ^∗^*P* < 0.05; ^∗∗^*P* < 0.01.

Next, neurosphere differentiation of four mEpiSC lines from naturally fertilized and NT embryos was performed, and the cell lines were compared in terms of morphology, gene expression, and differentiation capacities. All lines formed neurospheres when grown in neuronal differentiation media. However, major differences in terms of neurosphere size and abundance were evident (Figures 3A–C). The F-mEpiSCs formed more and bigger neurospheres compared with the NT-mEpiSCs. This effect was not a result of differences in the proliferation of undifferentiated mEpiSCs during neurosphere formation because undifferentiated F-mEpiSC and NT-mEpiSC lines proliferated at the same rate, as supported by the cell proliferation assay (Figures 3D,E).

### Quantification of Early Neurons Derived From F-mEpiSCs and NT-mEpiSCs

**FIGURE 4:**
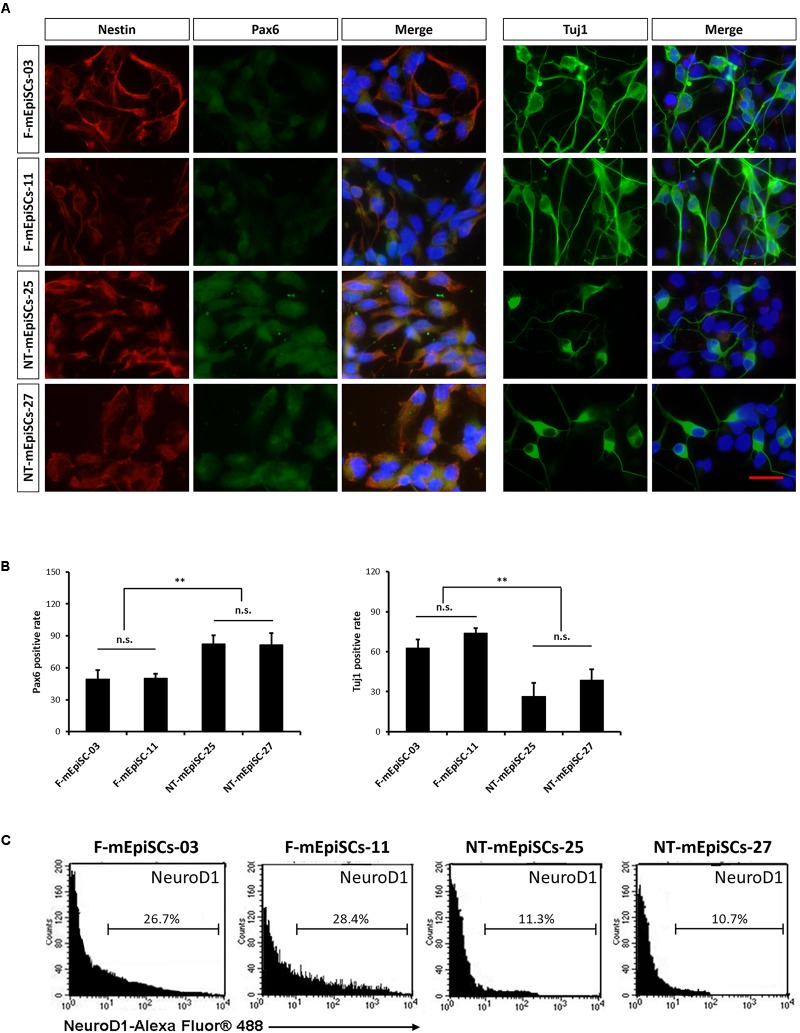
Quantification of early neurons derived from F-mEpiSCs and NT-mEpiSCs. **(A)** Immunofluorescence staining for Nestin, Pax6, and Tuj1 of early neurons derived from F-mEpiSCs and NT-mEpiSCs; nuclei are shown using DAPI **(B)** Quantification of Tuj1-positive and Pax6-positive cells based on Tuj1 and Pax6 immunofluorescence staining. **(C)** Flow cytometry analysis of F-EpiSCs and NT-EpiSCs stained for the neuronal marker NeuroD1. Mouse Neuro-2A cells served as positive control for the staining. Data were acquired for a minimum of 10 × 10^3^ events per sample using the FC500 flow cytometer and analyzed using the FlowJo software. Scale bar: 25 μm; n.s., *P* > 0.05; ^∗∗^*P* < 0.01.

**FIGURE 5:**
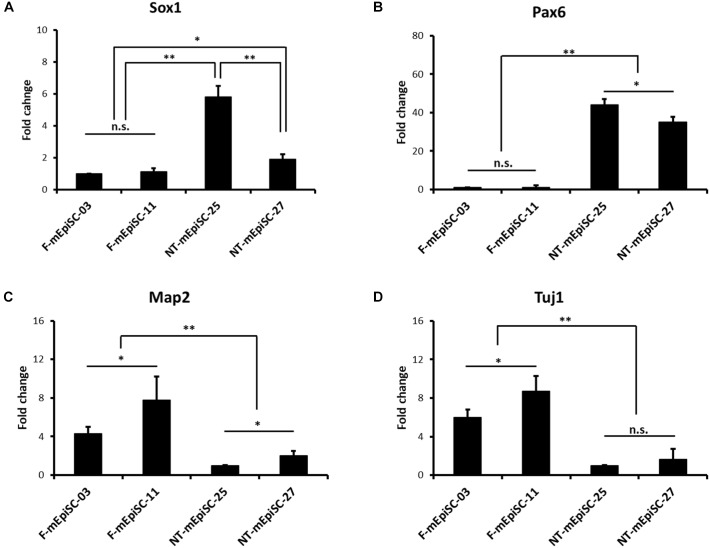
qPCR analysis of the neural-specific marker expression for early neurons derived from F-mEpiSCs and NT-mEpiSCs. **(A,B)** The expression of early neural markers Sox1 and Pax6 was significantly higher in NT-mEpiSC-derived early neurons than in F-mEpiSC-derived early neurons. **(C,D)** The expression of late neural markers Map2 and Tuj1 was significantly lower in NT-mEpiSC-derived early neurons than in F-mEpiSC-derived early neurons. A slower progress of neurogenesis *in vitro* of NT-mEpiSCs was indicated. GAPDH was used as endogenous control. n.s., *P* > 0.05; ^∗^*P* < 0.05; ^∗∗^*P* < 0.01.

Neurospheres from different lines were then dissociated into single cells, and fixed cell numbers were plated on poly L-ornithine/laminin-coated plates for 2 days for further differentiation. The neural cells were stained for neural markers Nestin, Pax6, and Tuj1 to characterize the differences between cell lines (Figure 4A). The percentages of positive cells were quantified in each cell line. Pax6-positive rates were significantly higher and Tuj1-positive rates were significantly lower in differentiated NT-mEpiSCs than in undifferentiated F-mEpiSCs (Figure 4B). The flow cytometry analysis reinforced these data as shown by the significantly lower proportion of NeuroD1-positive cells in differentiated NT-mEpiSCs than in differentiated F-mEpiSCs (Figure 4C). The qPCR analysis on differentiated mEpiSCs demonstrated that the transcript levels of early neural markers Sox1 and PAX6 were higher and the transcript levels of later neural markers Map2 and Tuj1 were lower in differentiated NT-mEpiSCs than in differentiated F-mEpiSCs (Figure 5). Sox1 and Pax6 are known to be upregulated in earlier stages of neuronal differentiation (Greber et al., 2010), with a low expression in neurons, suggesting that neurogenesis was slower in NT-mEpiSCs. Taken together, neuronal differentiation was more efficient and fast in F-mEpiSCs than in NT-mEpiSCs.

### Analysis of the Genes Relative to Neurogenesis Efficiency in Undifferentiated F-mEpiSCs and NT-mEpiSCs

**FIGURE 6:**
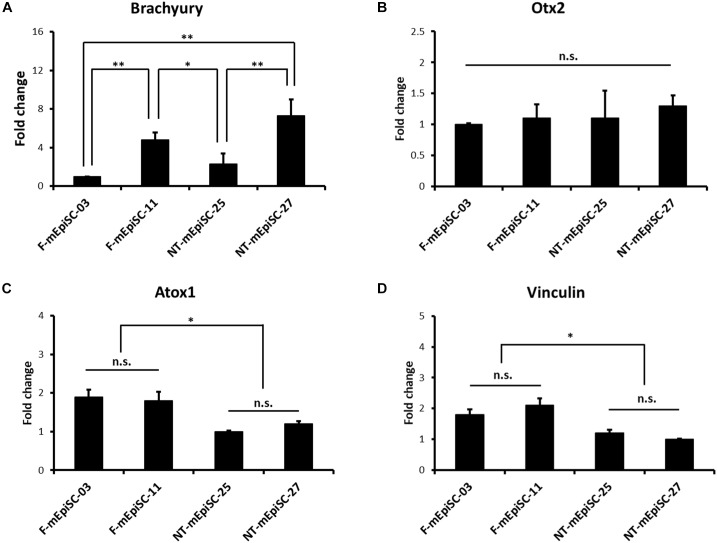
qPCR analysis of the genes expression relative to the neurogenesis efficiency in undifferentiated F-mEpiSCs and NT-mEpiSCs. **(A,B)** The expression of Brachyury and Otx2 in undifferentiated F-EpiSCs and NT-EpiSCs was not related with their differentiation potential. **(C,D)** The expression of Atox1 and Vinculin was significantly lower in undifferentiated NT-EpiSCs, which indicated the NT technique perhaps the underlying reason of neurogenesis hysteresis. GAPDH was used as endogenous control. n.s., *P* > 0.05; ^∗^*P* < 0.05; ^∗∗^*P* < 0.01.

The basal mRNA levels of mesendodermal markers Brachyury and Otx2 were compared in four mEpiSC lines in the undifferentiated state to investigate the causes for the variation in the neuronal differentiation capacity between F-mEpiSCs and NT-mEpiSCs (Figures 6A,B). Neuroectoderm precursor formation potential of EpiSCs is inversely correlated with mesendodermal marker expression (Bernemann et al., 2011). Differences in the expression levels of Brachyury were observed between the cell lines as shown by the higher expression in F-mEpiSCs-11 and NT-mEpiSCs-27. No major differences in Otx2 expression levels were observed. Importantly, the differences observed in Brachyury levels were not correlated with the differences in neuronal differentiation capacity of F-mEpiSCs and NT-mEpiSCs. Next, the basal mRNA levels of a metal sequestration protein antioxidant protein 1 (Atox1), which was capable of binding free metals to protect cells from the generation of reactive oxygen species, and a membrane-cytoskeletal protein Vinculin were analyzed in the four mEpiSC lines in the undifferentiated state (Figures 6C,D). Significant differences were shown in the expression levels for Atox1 and Vinculin between F-mEpiSCs and NT-mEpiSCs. These data reinforced the hypothesis that neuronal differentiation was more enhanced in F-mEpiSCs than in NT-mEpiSCs due to the alteration caused by the NT technique but not due to variations between cell lines.

## Discussion

Extensive studies over the past 10 years led to the hypothesis that the improper reprogramming and disruption of genomic imprinting was responsible for the developmental aberrations in NT (Dean et al., 2001). mEpiSCs have emerged as an extremely useful and relevant tool in this field. mEpiSCs, rather than mouse ESCs, derived from NT embryos display distinct genetic and epigenetic profiles, consistent with the disruption of genomic imprinting in NT blastocysts and animals (Maruotti et al., 2010). mEpiSCs provide a unique opportunity to investigate epigenetic aberrations associated with several developmental abnormalities or even embryo morbidity. Interestingly, most of the imprinted genes expressed in the brain were strongly downregulated in NT-mEpiSCs compared with F-mEpiSCs (Maruotti et al., 2010; Jouneau et al., 2012). It was predicted that NT-mEpiSCs differed from F-mEpiSCs in their *in vitro* neural differentiation capacity, and compelling evidence confirming this prediction was presented. The data reinforced the notion that mEpiSCs might serve a potential *in vitro* model to investigate early epigenetic and developmental processes.

In this study, mEpiSCs were successfully established from naturally fertilized and NT embryos and differentiated into early neurons using a standard protocol involving the formation of neurospheres in suspension and neuronal maturation on poly L-ornithine/laminin-coated plates. Four mEpiSC lines from the same genetic background, two F-mEpiSCs and two NT-mEpiSCs, were differentiated and compared in terms of morphology, gene expression, and differentiation capacities. Significant differences in neuronal differentiation were observed by size and abundance of neurospheres, number of neurons, and induced neuronal marker expression. Overall, this study demonstrated that neuronal differentiation was more efficient and fast in F-mEpiSCs than in NT-mEpiSCs. It showed that the expression of brain-specific transcripts was downregulated in NT-mEpiSCs than in F-mEpiSCs (Maruotti et al., 2010), for example, Ndn required for terminal neuronal differentiation (Aizawa et al., 1992; Takazaki et al., 2002); Peg3 important in early postnatal development of the brain (Broad et al., 2009); IMPACT implicated in GCN2-dependent learning and memory (Pereira et al., 2005; Bittencourt et al., 2008); and Pcsk9, a secreted protease that mediates the proper development of the nervous system in the brain (Kysenius et al., 2012). The downregulation of these genes led to abnormalities in the nervous system and delay in behavioral development. The findings of this study *in vitro* complemented those of *in vivo* studies.

Some studies showed that mEpiSC lines exhibited diverse potential to form neuroectoderm precursors, and this potential was inversely correlated with the expression patterns of the mesendodermal marker Brachyury in the undifferentiated state (Bernemann et al., 2011). Based on the findings of the present study, these effects could not be explained by the differential basal expression levels of mesodermal markers in the undifferentiated state. Several proteins dysregulated in NT-EpiSCs might be associated with the aberrant neuron-related phenotypes (Frohlich et al., 2013). For instance, the transcription factor Atox1 is associated with severe growth retardation, neurological problems, and congenital eye defects when disrupted (Hamza et al., 2001; Muller and Klomp, 2009). Also, the cytoskeletal protein Vinculin is implicated in neurological defects when inactivated, including the lack of midline fusion of the rostral neural tube and attenuation of nerve development (Xu et al., 1998). The pre-weaning behavioral development of cloned mice was delayed compared with that of naturally bred mice, with no subsequent differences in learning, memory, and motor abilities (Tamashiro et al., 2000). In the present study, the expression levels of Atox1 and Vinculin were significantly lower in NT-mEpiSCs than in F-mEpiSCs, leading to low early neurogenesis efficiency in NT-mEpiSCs.

Taken together, this study reported the neuronal differentiation abnormal phenotype *in vitro* in NT-mEpiSCs. The NT technique but not variations between cell lines could be the underlying reason of the delay in neuronal differentiation. This *in vitro* study also complemented a previous *in vivo* study showing that abnormalities in the nervous system and behavioral development were correlated with aberrations in NT-related epigenetic regulation. Further studies are needed to clarify the nature of NT-dependent effects and understand whether they are relevant for human-assisted reproductive technology.

## Ethics Statement

This study was carried out in accordance with the recommendations of the Guideline for the Care and Use of Laboratory Animals from the National Institute of Health. The protocols were specifically approved by the Committee on the Ethics of Animal Experiments of the Wenzhou Medical University.

## Author Contributions

DY and TL designed the experiment. TL and YZ performed the experiments. DY, TL, YZ, and YL analyzed the data. TL and DY wrote the paper. All authors were involved in revising the paper for important intellectual content, and gave final approval of the version to be published.

## Conflict of Interest Statement

The authors declare that the research was conducted in the absence of any commercial or financial relationships that could be construed as a potential conflict of interest.
