# Supplementary material for: Comparison of in vitro Neuronal Differentiation Capacity Between Mouse Epiblast Stem Cells Derived From Nuclear Transfer and Naturally Fertilized Embryos
Source: Front Mol Neurosci. 2018 Oct 30;11:392. doi: 10.3389/fnmol.2018.00392 (PMC6218595; doi:10.3389/fnmol.2018.00392)
Supplement: Supplementary file 1 [file Data_Sheet_1.PDF]

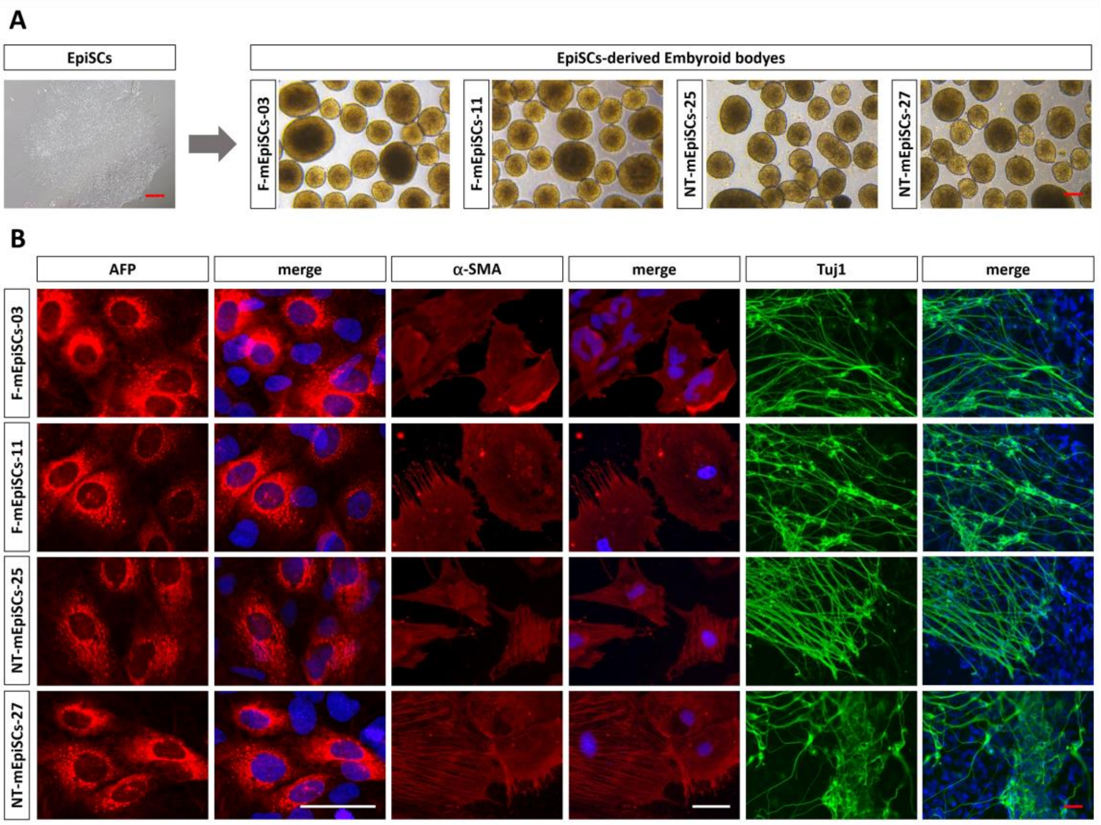

Supplementary Figure 1 (Fig. S1): Spontaneously differentiation of F-mEipSCs and NT-mEpiSCs to three germ layers toword embyroid body. (A) Morphology of embryoid bodies 7 days after F- and NT- EpiSCs were transfered to low attachment plates. (B) Immunofluorescence staining for endodermal marker Alpha Fetoprotein (AFP), the mesodermal marker alpha smooth muscle actin (α-SMA) and the ectodermal marker β-III tubulin (Tuj1) in Spontaneously differentiated F- and NT- mEpiSCs, nuclei are shown using DAPI. Scale bar: 100 μm.
